# Three-dimensional descriptive study of the pterygomaxillary region related to pterygoid implants: A retrospective study

**DOI:** 10.1038/s41598-019-52672-x

**Published:** 2019-11-07

**Authors:** Carmen Salinas-Goodier, Rosa Rojo, Jorge Murillo-González, Juan Carlos Prados-Frutos

**Affiliations:** 1 Department of Medicine and Surgery, Faculty of Health Sciences, Rey Juan Carlos University Avenida Atenas s/n, 28922 Alcorcón, Madrid Spain; 2 Department of Anatomy and Embryology, Faculty of Medicine, Complutense University, Avenida Complutense s/n, 28040 Madrid, Spain; 3 Dentistry Department, San Francisco de Quito University, Avenida Pampite s/n, 170157 Quito, Ecuador

**Keywords:** Anatomy, Oral anatomy

## Abstract

The objective of this study was to analyze three-dimensionally the morphological characteristics of the pterygomaxillary region related to pterygoid implants. Volume, height, width and bone density were studied in relation to age, sex and dental status. This retrospective observational study analyzed the CBCT of 52 hemi-maxillas three-dimensionally (females n = 28, males n = 24; dentate = 31, edentulous = 21). Patients were exposed between September 2009 and October 2014, and data collection was performed between November 2015 and May 2016. Bone density, volume, height and width were analyzed in various locations of the maxilla and pterygoid process, and the variables age, gender and dental status patients were compared. The results show that the mean width of the pterygomaxillary joint was 7.5 mm (SD 1.00 mm), mean height was 12.51 mm (SD 1,82 mm) and mean volume was 321.7 mm^3^ (SD 142.02 mm^3^). Statistically significant differences between dentate and edentulous patients were found, showing a higher osseous density in dentate patients in the pterygoid process (758.2, SD 106.8, 95% CI 729.2 to 787.3 GSD - Gray Scale Density - compared to 689.9, SD 107.3, 95% CI 660.8 to 719.1 GSD; *P* = 0.022). In the maxilla, density was statistically significant lower in female subjects (571.0, SD 74.1, 95% CI 594.9 to 645.4 GSD) than in male subjects (620.2, SD 93.8, 95% CI 594.4 to 645.4 GSD, *P* = 0.047). In conclusion, due to the significant variation in the morphological characteristics of the pterygomaxillary region among subjects, personalized pre-surgical radiological assessment should always be performed. Gender, age and dental status are critical factors as they significantly affect bone density in this region.

## Introduction

The loss of superior posterior teeth and the consequent alteration of the occlusion can lead to occlusal instability, deficiencies in the masticatory function and local bone loss, among other results^1^. When treating long-term edentulous patients, the surgeon may face a situation in which pneumatization of the maxillary sinus, resorption of the alveolar bone and insufficient osseous density occur together. The placement of implants in the posterior maxilla can become a challenge due to, among other factors, the poor osseous quantity and quality of the maxillary tuberosity^2^, and it has been demonstrated that implants have a higher propensity to fail under conditions of low-density bone^3^. Likewise, it has been proved that, with increasing age, the number of fibroblasts in the suture decreases and the suture and surrounding bone matures^4^, making bone quality and repair capacity decrease. Regarding the gender of the patient, inconclusive results have been obtained when examining the relationship between this variable and the bone density of the maxilla. One study did not found statistically significant differences when comparing genders^5^, while other assures that female subjects had significantly denser bone compared with male subjects^6^.

To avoid sinus lift surgeries or bone grafts, Tulasne^7^ first described the pterygoid implants in 1989 as an alternative to conventional dental implants. Unlike maxillary tuberosity implants, pterygoid implants anchor in cortical bone, allowing for a better primary stabilization, which is known to be a critical factor for long-term success^8^.

The pterygoid implants also present some inconveniences: the learning is convoluted compared to conventional dental implants, access for surgeons and oral rehabilitators is complex and serious injuries can occur in sectioning the maxillary artery or invading the pterygomaxillary fossa^2^.

For the placement of these implants, it is essential to have a thorough knowledge of the anatomy involved because nearby vital structures can be injured during surgery. Special attention should be paid to the internal maxillary artery, which crosses between 10 and 23.5 mm above the pterygomaxillary suture according to authors^9,10^. According to Candel *et al*.^9^, any bleeding observed in the preparation of the pterygoid implant bed comes from the irrigation of the pterygoid muscles and will be controlled once the implant is inserted. When placing a pterygoid implant, it is essential to pay attention to its proximity to the greater palatine canal and nerve^7,11^. There are other authors who believe that none important anatomical structure can be injured during the placement of these implants^12,13^.

One of the most important reasons and advantages for the use of pterygoid implants is the elimination of the need to perform sinus lift surgeries or bone grafts, therefore decreasing the morbility and shortening the treatment time, as it has been proved that the osseointegration of pterygoid implants can occur in only 2 to 3 months^7,14^. Unlike maxillary tuberosity implants, pterygoid implants anchor in cortical bone, allowing a better primary stabilization which is known to be a critical factor for long-term success^8^. From a prosthetic point of view it can minimize the time to rehabilitation and bypasses the need of a distal cantilever and also makes it possible to perform, if the patient meets the indication criteria, an immediate prosthetic load^15^. Another anatomical region in which dental implants can be anchored in advanced resorptions of the maxilla is in the zygoma, as it has been showed to have enough thickness and trabecular density^16^ to support occlusal forces, but compared to pterygoid implants, the learning curve for the surgical technique is greater and the potential surgical complications could be more severe.

Multiple studies have shown that pterygoid implants are a predictable treatment^14,16,17^ but few studies have described in a precise three-dimensional way the morphological characteristics of the pterygomaxillary region.

The aim of this study was to improve the knowledge of the morphological characteristics of the pterygomaxillary region by anatomical three-dimensional analysis to improve pterygoid implants surgical planning and thus improve long-term success rates. Volume, height, width and bone density of the pterygomaxillary joint were measured to assess whether age, sex or dental status significantly affected these variables.

## Methods

This study followed the Strengthening the Reporting of Observational Studies in Epidemiology (STROBE) recommendation guidelines^18^.

### Study design

A retrospective cross-sectional study was conducted at Rey Juan Carlos University (Madrid, Spain). The study received approval from the Rey Juan Carlos University Review Board and have been performed in accordance with the ethical standards of 1964 Declaration of Helsinki.

### Anonymization and analysis of the data

The collected data was anonymized for analysis by a data reduction technique conducted by an independent reviewer for the statistical treatment and evaluations of the variables (width, height and volume of the pterygomaxillary joint), ensuring the patients’ privacy.

### Participants

Twenty-eight patients, for a total of fifty-six sides (n = 56), between 27 and 71 years old were exposed between September 2009 and October 2014, and data collection was performed between November 2015 and May 2016. Cone beam computerized tomography (CBCT) from fifty-six hemi-maxillas were studied from patients who had undergone this radiological study for surgical reasons. Informed consent statement was obtained from all individual included in the study and the patients were informed about the potential risk of CBCT.

Inclusion criteria were patients between 27 and 71 years old, treated at the Rey Juan Carlos University for surgical purposes with a CBCT scan with a FOV (field of view) that included the entire pterygomaxillary complex. To include patients in this study data on gender and age had to be registered. The exclusion criteria were traumatic, cystic or tumor lesions in the study area; the presence of systemic diseases that partially or totally contraindicate implant treatment; skeletal deformities; images that were unclear or did not include the pterygomaxillary joint in its entirety; presence of any local bone pathology; patients with non-erupted third molars in the maxilla.

### Methodology

Radiographic explorations were performed with a CBCT (CS 8100 3D. Carestream). Default parameters for 3D were used: 80 kV, 2 mA, Field Of View (FOV) 8 × 9 cm, voxel size 150 µm. Patients were placed in a supine position. One cephalostat was placed stabilizing the head and palatal plane (ENA-ENP) perpendicular to the ground.

AMIRA® 6.0 software (AMIRA, Mercury Computer Systems, Berlin, Germany) was used to assess measure and create three-dimensional images of the pterygomaxillary region.

Two independent authors (J. A. M. G. and C. S. G.) performed all radiological measurements under the supervision of an expert oral and maxillofacial surgeon (J. C. P. F.).

### Sample size

In previous studies, the mean width and length of the pterygomaxillary joint was 8.5 mm (SD 1.9) and 12.9 mm (SD 2.7)^19^. The calculation was determined with a 99% confidence level, an accuracy of 1% and a variance of 3.61 and 7.29. A sample size of 24 and 48 subjects, respectively, was necessary. When the data collection of this study began, there were no published articles using the same references or methods as we did for the variables volume and bone density. Therefore, a preliminary pilot study was conducted to obtain this data. It was determined that the sample size for the bone density variable with a margin of error of less than 10%, was 52 hemi-maxillas.

### Analysis variables

**Figure 1:**
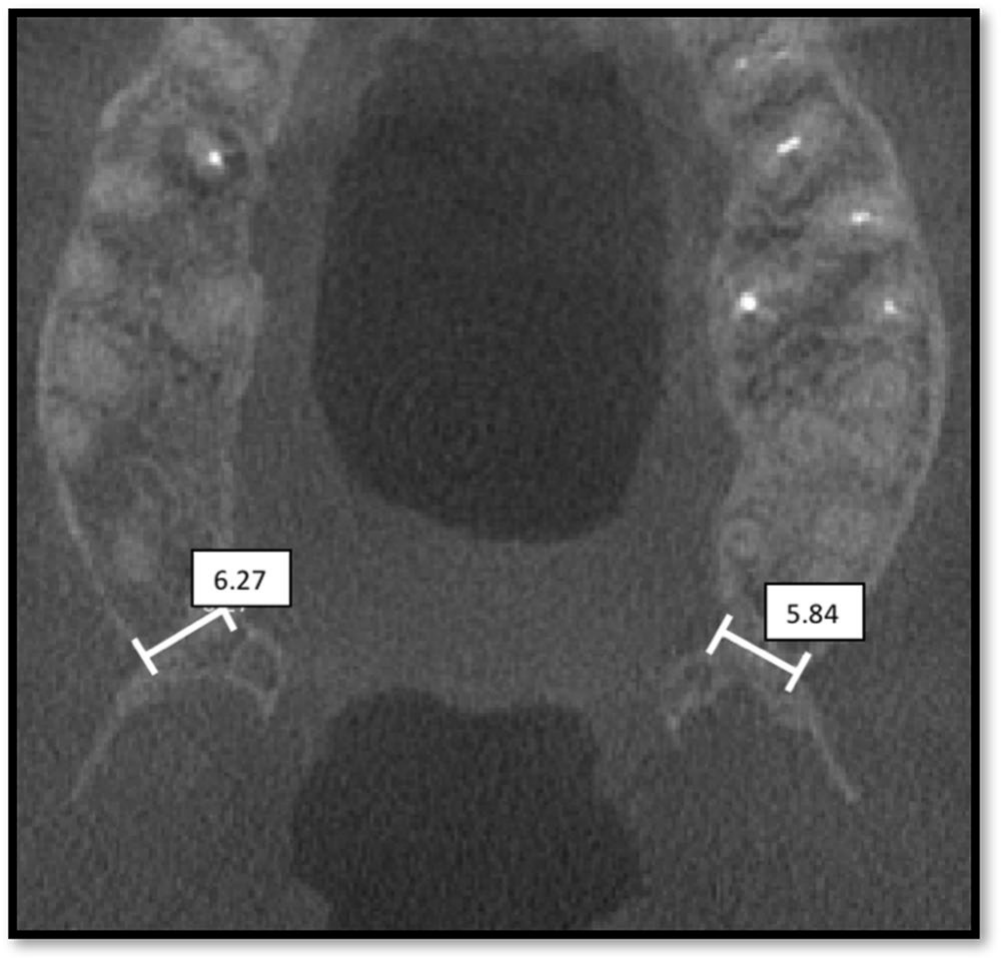
Image of the measurement of the width of the pterygomaxillary process: linear measurement in the transverse plane. The measurement was made in the first cut in which the tuberosity of the maxilla and the pterygoid process articulate completely.

**Figure 2:**
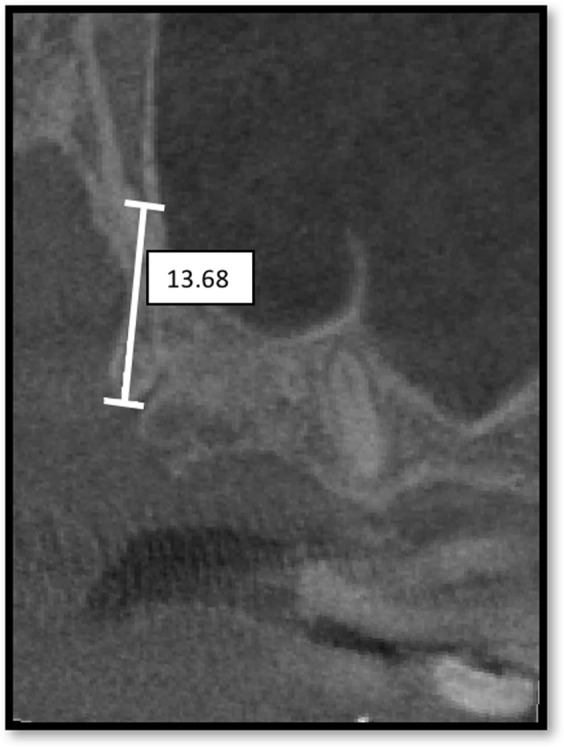
Image of the measurement of the height of the pterygomaxillary process: linear measurement in a modified sagittal plane. The measurement was made by selecting the most caudal point of the pterygomaxillary joint and the most cranial point to then perform a linear measurement.

The anatomical and radiological variables studied in this paper are described below. (1) Sex: male or female. (2) Age. (3) Side: right or left. (4) Joint width: this element was described as the joint width of the maxillary tuberosity and the pterygoid process in millimeters (mm), and the pyramidal process if present, measured on the first axial plane where a full joint contact was observed (Fig. 1). (5) Joint height: this element was described as the measurement in millimeters (mm) between the most caudal and most cranial points of the pterygomaxillary joint, which was considered the pterygomaxillary column (Fig. 2). (6) Bone volume: this element was described as the total bone volume in cubic millimeters (mm^3^). The area of study was delimited by the following limits: the posterior wall, the maxillary sinus, the pterygoid and scaphoid process, the inferior limit of the pterygomaxillary fossa and the separation of the tuberosity and pterygoid process. (7) Bone density: nine points were measured in total, three points at the inferior part of the pterygomaxillary column, three points at the superior part and three in a medium zone equidistant from the two previously described. Each of these areas was measured at the anterior limit (posterior sinus wall), the joint between the maxillae and the pterygoid/pyramidal process and the posterior limit (scaphoid or pterygoid fossae). As surgical radiological assessment was proceeded by CBCT, bone density was measured in gray values (GSD). According to a previous study^20^, gray values from CBCT could be used to assess bone quality and had been used previously in various studies^21–23^. (8) Dental status: dentate/edentulous: if the second or third upper molars were present, the patient was considered dentate. If none of these molars were present, the patient was considered edentulous.

### Statistical analysis

Two observers (J. A. M. G. and C. S. G.) performed the measurements. Student’s paired t test, non-parametric Wilcoxon contrast and the intraclass correlation coefficient (ICC) were used to analyze agreement between observers in all the variables.

A descriptive statistical analysis was performed using IBM SPSS^®^ version 19.0 statistical package (IBM Corp., Armonk, NY, USA), in which the variables of joint width, joint height, bone volume and bone density were crossed with genre, age, side and dentate/edentulous status.

First, normality was tested for all the variables with Kolmogorov-Smirnov and Shapiro-Wilk tests. The Student’s T-Test was applied if normality was met, and a non-parametric U Mann-Whitney test was used if it was not met. To evaluate concordance between measurement and age, Pearson’s correlation coefficient, or Spearman’s non-parametric correlation when applicable, was used. Results were considered statistically significant when *P* < 0.05.

The required sample size was calculated to obtain precise volume and linear measurements so that the confidence interval did not vary by more than 10% of the corresponding mean value and presented a confidence level of 95%.

## Results

**Figure 3:**
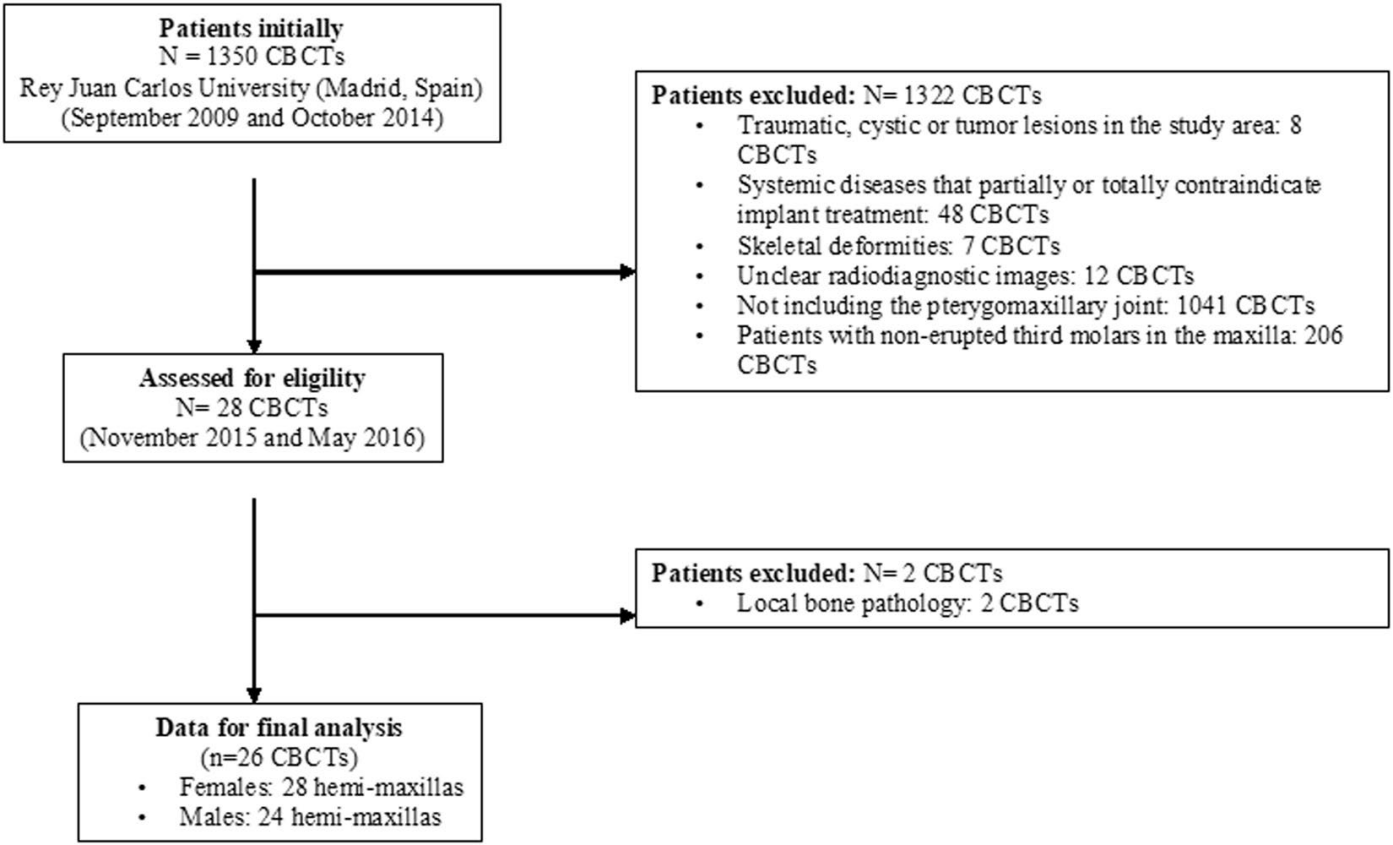
Flow chart showing the selection of this study sample.

Of 28 CBCTs available (56 sides), 26 CBCTs (52 sides) met the inclusion criteria (Fig. 3). The two CBCTs that were not included were due to the presence of bone pathology in the study area. Females represented 53.8% (n = 28) of the study sample, and 46.2% (n = 24) were males. The subjects’ mean age was 49.7 (SD 10.3). Right and left sides were equally distributed representing 50% (n = 26) of the sample each.

The intraclass correlation coefficient (ICC) varied from 0.82 (95% CI = 0.60 to 0.92) and 0.99 (95% CI = 0.81 to 0.94), which is considered excellent^24^.

Normality tests were applied, and all the variables but “volume” met this condition. Non-parametric tests were used when necessary. No missing data was identified in any of the variables.

The mean width of the joint was 7.51 mm (SD 1.0 mm, 95% CI 7.14 to 7.78 mm); mean width for females was 7.51 mm (SD 0.82, 95% CI 7.29 to 7.73 mm) and for males 8.07 mm (SD 1.23, 95% CI 7.74 to 8.40 mm). Mean height was 12.5 mm (SD 1.8 mm, 95% CI 12.01 to 12.99 mm); mean height for females was 12.55 mm (SD 1.60, 95% CI 12.12 to 12.98 mm) and for males 12.72 mm (SD 2.06, 95% CI 12.16 to 13.28 mm). Mean volume was 321.76 mm^3^ (SD 142.02 mm^3^, 95% CI 283.2 to 360.4 mm^3^); mean volume for females was 395.39 mm^3^ (SD 177.13, 95% CI 347.25 to 443.53 mm^3^) and for males 274.18 mm^3^ (SD 50.11, 95% CI 260.56 to 287.80 mm^3^).

Age, sex or dental status had no impact on the length or width of the pterygomaxillary joint. According to our study, these variables would not have an effect on the volume of the pterygomaxillary region either, however, for this variable a larger sample is needed to contrast the data.

Bone density values can be observed in Table 1. Considering male subjects and female subjects together, for the maxillary tuberosity density in the most caudal measurement, Pearson’s correlation index was −0.34, which was statistically significant (*P* = 0.015) and can be interpreted as older patients having less density in the tuberosity at its lowest point.

**Table 1:** Bone densities depending on the anatomical region and separated by gender.

	Male		Female	
	Mean ± SD	CI (95%)	Mean ± SD	CI (95%)
**Superior limit**				
AL	471.0 ± 112.9	440.3–501.7	484.4 ± 118.3	452.3–516.6
PMJ	571.8* ± 74.1	551.7–591.9	620.2* ± 93.3	594.8–645.5
PL	703.4 ± 115.4	672.0–734.8	730.1 ± 108.0	700.8–759.5
**Middle section**				
AL	434.7 ± 90.1	410.2–459.2	426.1 ± 77.5	405.0–447.1
PMJ	602.5 ± 88.2	578.5–626.5	554.1 ± 101.7	526.5–581.7
PL	726.8* ± 82.9	703.7–748.7	668.3* ± 105.5	639.7–697.0
**Inferior limit**				
AL	373.8 ± 59.6	357.6–390.0	367.3 ± 81.2	345.2–389.3
PMJ	599.0 ± 68.0	580.5–617.5	608.0 ± 116.3	577.3–640.5
PL	692.9 ± 85.8	669.6–716.3	691.7 ± 91.3	666.9–716.5

AL, anterior limit; PMJ, pterygomaxillary joint; PL, posterior limit.

*Statistically significant.

Dentate subjects represented 59.6% of the study sample (n = 31), and 40.4% (n = 21) were edentulous. When dentate and edentulous patients were studied separately, in dentate patients, the bone density of the middle section of the pterygomaxillary fissure increased with age (Pearson’s correlation = 0.46, *P* = 0.01). Also, in dentate patients, a statistically significant difference was observed between males’ and females’ middle sections of the pterygoid process (males: 734.7 GSD, SD 91.9 GSD, 95% CI = 709.7 to 759.7; females: 649.7 GSD, SD 105.1 GSD, 95% CI = 621.1 to 678.2 GSD; *P* = 0.026). In edentulous patients, density in the maxillary tuberosity’s middle section was significantly lower in female subjects (559.7 GSD, SD 85.4 GSD, 95% CI = 536.5 to 582.9 GSD) than in male subjects (631.1 GSD, SD 68.9 GSD, 95% CI = 612.4 to 649.8; *P* = 0.047). Also, the cancellous bone density of the maxillary tuberosity in male subjects was significantly higher (448.1 GSD, SD 74.6 GSD, 95% CI = 427.8 to 468.4) than in female subjects (366.1 GSD, 85.9 GSD, 95% CI = 342.8 to 389.4; *P* = 0.031).

## Discussion

This research shows three critical factors affect osseous density locally: gender (female subjects had statistically less density), age (density decreased as age progressed) and dental status (edentulous patients presented an inferior density).

In a study performed by Lee *et al*.^25^, the height of the pterygopalatine suture has been reported to be 13.1 mm, which is in concordance with the measurement in our study, 12.5 mm (SD 1.8 mm). Studies have suggested that implants measuring 13.0 to 18.0 mm could be appropriated to ensure an engagement in the cortical bone of the pterygoid process^17,26^.

The width of the pterygomaxillary joint has been previously studied in articles related to surgical techniques such as Le Fort I osteotomies^27,28^. However, we have not been able to find research focused on implantology that evaluates this characteristic. Our findings are slightly shorter than those measured by Dadwal *et al*.^27^ and Chin *et al*.^28^. This may be due to the differences in the measurement method, since in our study the measurement was always made of the most caudal region and in the previously mentioned articles they do not specify at what point of the pterygopalatine fissure the measurements were made or how they considered the exact point of measurement.

We have not found other studies in the literature that measure the volume of the maxillary tuberosity, therefore, we cannot compare the results.

Regarding bone densities, Rodríguez *et al*.^29^ reported an average of 307.4 GSD (SD 155.9 GSD) in the maxillary tuberosity and 632.0 GSD (SD 209.7 GSD) in the pterygoid area. Our measurements were slightly higher for both maxillary and pterygoid region (370.3 GSD, SD 71.4 GSD; and 692.4 GSD, SD 85.5 GSD; respectively), which could be due to mean age, which is not specified in Rodríguez’s study. In this study, bone density in the pterygoid area was 139.2% higher than in the tuberosity area, proving that it is an area of better choice for the anchorage of the implant head^29^. It has been proven that implants in type I and II bone had a higher survival rate and could support sand with a higher occlusal load for which type IV bone was unsuitable^30^, which supports our recommendation of using pterygoid implants for the rehabilitation of the posterior maxillae.

In our study, it was observed a statistically significant difference at the upper area on the pterygoid process, where dentate subjects presented a density of 758.2 GSD (SD 106.8 GSD. 95% CI 729.2 to 787.3 GSD) and edentulous patients presented 686.9 GSD (SD 107.3, 95% CI 657.8 to 716.1 GSD) (p < 0.05). This is believed to be explained because dentate patients have proven to have a greater muscular strength which develops into a mayor osseous density. A minor electromyographic activity of the masticatory muscles is related to the deterioration of the dental status^31,32^. Also, the middle section of the pterigomaxilar column had a greater density along with age; the older the subject was, the greater the density of the bone (Pearson’s correlation = 0.46, p = 0.01). This is also believed to be a result of muscular strength^33^. The osseous density of this middle section of the pterygoid process is important because is one of the main sites of anchorage of the pterygoid implants. These implants are anchored in the pterygoid plate of the sphenoid bone, moving backwards and upwards through the maxillary tuberosity and palatine bone^13,15,34^. It was observed that an implant placed in the pterigomaxilar area with an angulation on 45 degrees would increase a mean of 8–9 mm the bone to implant contact^13^.

As for the angulation of the pterygoid implants with respect to the Frankfort plane, there is no consensus. Different authors defend their placement with an approximate angle of 45°^13,15,35^ while in more recent studies their placement is defended at an angle of 70°^27–35^. In a study developed by Rodríguez *et al*.^36^, where 454 implants were placed in 392 patients, the mean angulation was 70.4° ± 7.2°. Anatomical studies support this technique: the average inclination of the bone pillar composed of the pterygoid process and the tuberosity of the maxilla is between 67.3° and 75.1° in relation to the Frankfort plane^37^. Therefore, previous studies that suggested placing the pterygoid implants with an approximate inclination of 45° with respect to the Frankfurt plane would be relegated^13,15,34^. This traditional angulation of the pterygoid implants has different disadvantages, the main one being that the transmission of the occlusal forces does not occur in an axial direction, and may compromise the long-term result of the prosthetic rehabilitation^36^. An inclination of 45° decreases its load capacity by up to 50% with respect to the same implant placed at 90°^34^. Therefore, a 20 mm pterygoid implant with an angle of 45° would have the same occlusal loading capacity as a 10 mm implant placed at 90° ^36^.

Pterygoid implants were first described by Tulasne^2^ as a reliable method for the rehabilitation of the atrophied posterior maxilla. It was described as an alternative to rehabilitate the atrophic maxilla avoiding the need for previous surgeries such as maxillary sinus augmentation or alternative bone graft^9,15^. In 1992, Tulasne described a success rate of 80%^7^, nonetheless during the past years several studies have reported a much higher success rate, reporting from 90.7% to 99% of success^9,14,16,17,38,39^.

These implants are proven to compensate for the posterior maxilla’s poor osseous quality by anchoring themselves to the pterygoid process’s cortical bone^2,7,21^. Dental implants placed in the tuberosity have also been widely described^34,40,41^, as it is possible to place an implant completely and solely in the maxillary tuberosity if the bone’s quantity and quality are adequate, but is also well-known that in most of the cases, this anatomical region is conformed by Type III or Type IV cancellous bone^21^. Among others, loss of the dental implants is related to low-primary stability, which is at the same time related to bone density^40^. This research confirms that the tuberosity has a very low bone density compared to the pterygoid region, agreeing with previous studies^9^. It has also been shown that increasing dental implant length plays a fundamental role in increasing dental implant primary stability^42^.

In patients with atrophic maxillae who are not subsidiaries of receiving bone grafts or short implants, the placement of zygomatic implants should also be assessed^43^. The main indication of these implants is the rehabilitation of completely edentulous patients, with severe pneumatization of the maxillary sinuses and severe resorption of the posterior alveolar ridge^43^. The volume and extension of the maxillary sinus along with the angulation of the anterior wall will determine the position of the most distal implant and its angulation. This, unless pterygoid implants are also placed, will define the distal extension of the prosthesis^43,44^. The survival rate of this type of implants is high (97.86% in a 36-month follow-up period), according to the systematic review by Goiato *et al*.^45^. The placement of zygomatic implants is complex due to the intricate anatomy of the maxillofacial region and the greater length of these implants (from 30 to 52.5 mm)^44^. Although some authors argue that the surgical and postoperative complications of these implants are infrequent^44^, if any of them appear they can be serious. Among the main complications are: rhinosinuisitis^46^, lack of osseointegration^47^, soft tissue infection^48,49^, paraesthesia^48,49^ and oroantral fistula^48,50^.

For the placement of these implants, it is imperative to have a thorough knowledge of the anatomy involved because nearby vital structures can be injured during surgery. Special attention should be paid to the internal maxillary artery^7,9–11^.

## Conclusions

Pterygomaxillary region morphology has a wide range of variation between individuals, and every time a pterygoid implant is planned to be placed in this area, a personalized pre-surgical radiological assessment should be performed. Various factors such as dental status (edentulous patients presented an inferior density), age (density decreased as age progressed) and gender (female subjects had statistically less density) have an impact on bone density in the pterygomaxillary region. Nevertheless, it has been shown that the bone density of the pterygoid process is always greater than the bone density of the maxillary tuberosity and, therefore, it should be the choice for implant anchoring in atrophic maxillae.
